# A Randomized Comparison of two Short Intensive Chemotherapy Regimens in Children and Young Adults With Osteosarcoma: Results in Patients With Metastases: A Study of the European Osteosarcoma Intergroup

**DOI:** 10.1080/13577149778245

**Published:** 1997-12

**Authors:** Vivien H. C. Bramwell, Marion V. Burgers, Robert L. Souhami, Antonie H. M. Taminiau, Jan W. Van Der Eijken, Alan W. Craft, Archie J. Malcolm, Barbara Uscinska, Anna L. Kirkpatrick, David Machin, Martine M. Van Glabbeke

**Affiliations:** 1 London Regional Cancer Centre 790 Commissioners Road East London Ontario N6A 4L6 Canada; 2 Netherlands Cancer Institute Amsterdam The Netherlands; 3 University College & Middlesex School of Medicine London UK; 4 Academisch Ziekenhuis Leiden The Netherlands; 5 Onze Lieve Vrouvwe Gasthuis Amsterdam The Netherlands; 6 Royal Victoria Infirmary Newcastle upon Tyne UK; 7 MRC Cancer Trials Office Cambridge UK; 8 EORTC Data Centre Brussels Belgium

## Abstract

*Purpose*. To report the outcome of 37 patients with metastatic osteosarcoma entered into a large randomized trial (EOI 80831/MRC B002) comparing two different regimens of chemotherapy in patients with osteosarcoma.

*Methods*. Patients with biopsy-proven osteosarcoma localized and metastatic, age 40 years or younger, were randomized to receive either two-drug treatment with doxorubicin/cisplatin (DOX 25 mg m^-2^ day^-1^ × 3 + DDP 100 mg m^-2^ on day 1 q 3 weeks × 6 courses) or three-drug treatment comprising high-dose methotrexate (HDMTX 8 mg m^-2^ administered every 4.5 weeks × 4 courses) given 10 days before DOX/DDP.

*Results*. Twenty-four patients with metastatic disease received the two-drug arm treatment and 13 received three-drug treatment. Despite chance imbalance in numbers, there were no major differences in age, sex, primary site or performance status. Baseline alkaline phosphatase (AP) was elevated more frequently (96 vs 42%) in the two-drug arm. Twenty-one of 24 patients in the two-drug arm and 11/13 patients in the three-drug arm had evaluable primary tumors concurrent with metastases. Respective clinical response rates for the two- and three-drug arms were 48% and 40% for primary tumors, and 33% and 55% for metastases. Respective survivals at 2 and 4 years were 36% and 9% for the two-drug arm, and 69% and 52% for the three-drug arm, and survival was better for patients with normal AP at presentation. When adjusted for AP, survival was not significantly different between the two treatments (hazard ratio 0.52, 95% confidence interval 0.22–1.23, *p* = 0.14). There were three long-term survivors among the metastatic patients, all of whom received the three-drug therapy.

*Discussion*. It is likely that random bias in the population (small numbers, imbalance in size of groups, uneven distribution of AP) accounts for the difference in outcome favoring the three-drug treatment in patients with metastatic disease. More reliance can be placed on the finding that disease-free and overall survival in the adjuvant component of this study (Bramwell *et al., J Clin Oncol* 1992; 10: 1579–91) were better after two-drug treatment.


*Sarcoma* (1997) 1, 155–160

CARFAX

ORIGINAL ARTICLE

### A randomized comparison of two short intensive chemotherapy regimens in children and young adults with osteosarcoma: results in patients with metastases: a study of the European Osteosarcoma Intergroup

VIVIEN H. C. BRAMWELL,[1] MARION V. BURGERS,[2] ROBERT L. SOUHAMI,[3] ANTONIE H. M. TAMINIAU,[4] JAN W. VAN DER EIJKEN,[5] ALAN W. CRAFT,[6] ARCHIE J. MALCOLM,[6] BARBARA USCINSKA,[7] ANNA L. KIRKPATRICK,[8] DAVID MACHIN,[7] & MARTINE M. VAN GLABBEKE[8]

[1]*London Regional Cancer Centre, London, Canada,* [2]*Netherlands Cancer Institute, Amsterdam, The Netherlands,* [3]*University College & Middlesex School of Medicine, London, UK,* [4]*Academisch Ziekenhuis, Leiden, The Netherlands,* [5]*Onze Lieve Vrouvwe Gasthuis, Amsterdam, The Netherlands,* [6]*Royal Victoria Infirmary, Newcastle upon Tyne, UK,* [7]*MRC Cancer Trials Office, Cambridge, UK &* [8]*EORTC Data Centre, Brussels, Belgium*

**Abstract**

*Purpose.* To report the outcome of 37 patients with metastatic osteosarcoma entered into a large randomized trial (EOI 80831/MRC B002) comparing two different regimens of chemotherapy in patients with osteosarcoma.

*Methods.* Patients with biopsy-proven osteosarcoma localized and metastatic, age 40 years or younger, were randomized to receive either two-drug treatment with doxorubicin/cisplatin (DOX 25 mg $m^{-2}$ $day^{-1}$ $\times 3$ + DDP 100 mg $m^{-2}$ on day 1 q 3 weeks $\times 6$ courses) or three-drug treatment comprising high-dose methotrexate (HDMTX 8 mg $m^{-2}$ administered every $4\frac{1}{2}$ weeks $\times 4$ courses) given 10 days before DOX/DDP.

*Results.* Twenty-four patients with metastatic disease received the two-drug arm treatment and 13 received three-drug treatment. Despite chance imbalance in numbers, there were no major differences in age, sex, primary site or performance status. Baseline alkaline phosphatase (AP) was elevated more frequently (96 vs 42%) in the two-drug arm. Twenty-one of 24 patients in the two-drug arm and 11/13 patients in the three-drug arm had evaluable primary tumors concurrent with metastases. Respective clinical response rates for the two- and three-drug arms were 48% and 40% for primary tumors, and 33% and 55% for metastases. Respective survivals at 2 and 4 years were 36% and 9% for the two-drug arm, and 69% and 52% for the three-drug arm, and survival was better for patients with normal AP at presentation. When adjusted for AP, survival was not significantly different between the two treatments (hazard ratio 0.52, 95% confidence interval 0.22–1.23, $p = 0.14$). There were three long-term survivors among the metastatic patients, all of whom received the three-drug therapy.

*Discussion.* It is likely that random bias in the population (small numbers, imbalance in size of groups, uneven distribution of AP) accounts for the difference in outcome favoring the three-drug treatment in patients with metastatic disease. More reliance can be placed on the finding that disease-free and overall survival in the adjuvant component of this study (Bramwell *et al.*, *J Clin Oncol* 1992; 10:1579–91) were better after two-drug treatment.

**Key words:** *osteosarcoma, chemotherapy, metastases.*

### Introduction

The most common type of osteosarcoma is the so-called 'classical variety', which occurs in the limbs of children and young adults. Until the introduction of intensive adjuvant chemotherapy, the outlook after surgery alone was extremely poor[1] but with modern multi-agent chemotherapy, 5-year survival figures for non-metastatic cases range between 40 and 80%.[2]

Unfortunately, between 15 and 20% of patients with osteosarcoma will present with clinically detectable metastases, a proportion that has increased with sophisticated methods of detection, such as computed tomography (CT) scanning of the lungs. It is common to treat these patients aggressively with intensive multi-agent chemotherapy, followed by resection of metastases particularly if these are confined to the lung, but results are poor.[3,4]

As determined by measurable objective response, chemotherapy has always produced rather unim-

Correspondence to: V. H. C. Bramwell, London Regional Cancer Centre, 790 Commissioners Road, East, London, Ontario, Canada N6A 4L6. Tel: +1 519 685 8640; Fax: +1 519 685 8624; E-mail: vbramwell@lrcc.on.ca.

1357-714X/97/030155-06 $9.00 © 1997 Carfax Publishing Ltd pressive results in metastatic osteosarcoma.[5] Early data showed overall response rates for single agents such as doxorubicin, cisplatinum, high-dose methotrexate and ifosfamide in the range 15–25% with even lower response rates 8–15% for agents such as cyclophosphamide, actinomycin D, melphalan, dacarbazine and mitomycin C. Similarly, although in some studies, response rates as high as 50–80% have been reported for combination chemotherapy, these results have usually been obtained in very small series of patients. More frequently, response rates have been in the range 25–50%. Inclusion of patients with very tiny lung metastases in more recent series may make evaluation unreliable. Response may also be underestimated, as it is not uncommon for lesions, both primary and secondary, to calcify but not shrink on chemotherapy. Subsequent resection sometimes reveals these tumors to be largely necrotic and/or fibrotic with calcification, and in some cases unresected nodules may never entirely resolve.

In 1983, the European Osteosarcoma Intergroup initiated a randomized phase II trial in which two short intensive cytotoxic regimens were to be compared for response and toxicity. Treatment in one arm was an intensive doxorubicin/cisplatin (DOX/DDP) regimen, and in the other arm the same drugs were given alternately with high-dose methotrexate (HDMTX). These were chosen because the role of HDMTX in patients treated with other drugs known to be highly effective in osteosarcoma was controversial. The study recruited patients rapidly, and the manageability and early efficacy of the regimens, judged by clinical response, was such that the study was expanded to a formal phase III trial in which survival was the main endpoint. Results of this protocol in non-metastatic patients who received neo-adjuvant and adjuvant chemotherapy have been reported previously.[6] As this protocol was originally designed to evaluate response and toxicity, patients presenting with initial metastases, axial or locally recurrent tumors were also eligible for the study, and this report describes the outcome for the patients with metastatic disease.

### Patients and methods

### *Study population*

Patients with biopsy-proven high-grade osteosarcoma, who were aged 40 years or younger, were eligible for this study. To be evaluable for the current report, patients had to have distant metastases. The latter group included patients presenting with a primary tumor and concurrent metastases, and those relapsing with metastases after previous treatment of a primary tumor by surgery only. At entry, patients were required to have adequate renal function (serum creatinine $\leq 150$ $\mu$mol $l^{-1}$), hepatic function (serum bilirubin $\leq 20$ $\mu$mol $l^{-1}$) and bone marrow reserve (white blood count $\geq 4 \times 10^9$/l, platelets $\geq 100 \times 10^9$/l). Informed consent was obtained according to local institutional policies. Patients who had received previous chemotherapy or radiotherapy were not eligible, nor were those with other malignancies or concomitant disease that prevented intensive chemotherapy. Non-classic forms of osteosarcoma such as parosteal, periosteal, Pagetoid and post-irradiation tumors were not included.

### *Trial design and therapeutic regimens*

In this subgroup of patients with metastatic tumors, the objectives were to assess clinical and radiological response and survival. Toxicity was described in the previous report[6] and was not quantitatively different in this group of patients. The method of randomization has been described previously.[6]

The treatments were designed to be of equal duration so that the planned dose intensities of DOX plus DDP were different between the two arms. The two-drug regimen comprised six courses of DOX 25 mg $m^{-2}$ on days 1–3 given by intravenous (iv) bolus and DDP 100 mg $m^{-2}$ on day 1 given by 24-h iv infusion at 3-week intervals. The three-drug regimen used the same DOX/DDP chemotherapy, but only four courses were given, each preceded 10 days earlier by HDMTX 8 g $m^{-2}$ by 4-h iv infusion with appropriate leucovorin (LV) rescue: LV 12 mg $m^{-2}$ iv or 15 mg $m^{-2}$ for 6 h orally began 24 h after the start of MTX infusion for a total of 10 doses. Serum MTX levels were measured at 24 and 48 h. If the 48-h level was below $10^{-7}$ mol $l^{-1}$, no additional LV rescue was required. Levels higher than this required adjustment of the LV dose or duration according to a nomogram.[7] Details of the hydration regimen, dose modifications, and pretreatment and follow-up investigations are given in the previous report.[6]

### *Response criteria: primary*

*Clinical.* The circumference of the limb and primary tumor length were recorded, and the treating physician was asked to categorize the response into complete remission, improvement, no change and progressive disease. Accurate clinical measurement in two perpendicular diameters was not felt to be possible because changes in tumor masses could be obscured by factors such as post-biopsy edema, hematoma and muscle wasting. Components such as pain relief and reduction in inflammatory signs were taken into account, but the final categorization was essentially subjective.

*Radiologic.* A number of factors were considered including the length and width of the tumor on radiographs and CT, increased ossification and, in some cases, reduced vascularity. Although central radiologic review was attempted, differences in technology, particularly for CT, made this a difficult exercise and it was abandoned. Thus, radiologic response categorization into complete remission, improvement, no change and progressive disease was also subjective.

### *Response criteria: metastases*

Response of measurable metastatic disease was assessed as follows.

*Complete response.* Disappearance of all symptoms and signs of tumor determined by two observations not less than 4 weeks apart.

*Partial response.* Decrease of 50% or more in the sum of the products of measured lesions determined by two observations not less than 4 weeks apart, and no simultaneous increase in the size of any lesion or the appearance of new lesions. Non-measurable lesions had to remain stable or regress for this category.

*Stable disease.* Steady state of response less than partial remission (i.e. $< 50\%$ decrease in sum of the products of measured lesions), or progression less than progressive disease, of at least 6 weeks duration. There could be no appearance of new lesions for this category.

*Progressive disease.* Unequivocal increase of at least 25% in the sum of the products of measured lesions, or appearance of significant new lesions.

### *Statistical methods*

Response to treatment is presented in contingency tables. Response to treatment being expressed as ordered categorical variables (see earlier), comparisons between response rates have been performed using the $\chi^2$ test for trend for ordered categorical data.[8] Additionally, the Fisher exact test was performed to compare overall response rates (rate of complete plus partial responses). Exact methods were used to compute confidence intervals (CI) of overall response rates and of their difference. The probability of survival was calculated using the Kaplan–Meier estimate,[9] and comparison between therapeutic groups used the log-rank test. The test was subsequently adjusted[10] for the initial level of alkaline phosphatase (AP).

### Results

Between January 1983 and December 1986, 307 patients were registered in the entire study. As it proved impossible to obtain sufficient data to determine eligibility and survival for patients who were entered by one cooperative group and two additional centres, the entire set (30 patients) for these institutions was removed. There were five more patients than documented in a previous report of neo-adjuvant/adjuvant cases,[6] as the excluded group now contains patients with metastatic disease. Two hundred and twenty-eight adjuvant and neo-adjuvant cases have been previously reported.[6] Seven patients with axial and three with locally recurrent tumors, not associated with metastases, are not considered further in this report.

Of 39 patients with metastatic disease, two patients who received the three-drug regimen were considered to be ineligible. One patient with a primary tumor in the upper humerus was originally thought, on angiography, to have involved axillary lymph nodes, but this could not be confirmed at subsequent review. The second patient had received prior chemotherapy. Thus, 37 patients with metastatic disease at presentation are the subject of this report.

The clinical characteristics of the metastatic cases are shown in Table 1. Twenty-four cases were randomized to receive the two-drug regimen and 13 to receive the three-drug regimen. Allowing for small numbers and a chance excess of cases in the two-drug arm, there were no major differences in characteristics such as age, sex, primary site and performance status according to treatment arm. The median age was 18 years. Elevated levels of serum AP at presentation were observed more frequently (96% vs 42%) in the two-drug arm. In 21/24 patients in the two-drug arm and 11/13 patients in the three-drug arm, metastases were present concurrent with the primary tumor.

Eleven patients (46%) completed the full course of six treatment cycles on the two-drug arm. Progression of disease occurred in nine patients after one (one patient), two (one patient), three (four patients), four (two patients) and five (one patient) cycles. Two patients refused treatment after three and five cycles, and two patients stopped after five cycles because of liver function abnormalities and impaired renal function, respectively. In contrast, on the three-drug regimen, only two patients failed to complete the full course of four cycles of chemotherapy, terminating after one and three cycles, in both cases due to disease progression. Eight patients received the four planned cycles and three patients with responding disease received one to three additional cycles.

In the two-drug arm, 10/21 patients had resection of the primary tumor. In eight cases this was performed after three cycles of chemotherapy, as recommended in the protocol. In one patient, who did not receive the last two cycles because of progressive lung metastases, an above-knee amputation was performed 6 months later. A second patient, who achieved partial remission of both an upper humeral primary and metastases, underwent conservative surgery with insertion of a prosthesis after six cycles of chemotherapy. Five patients who had

**Table 1.** *Clinical characteristics, patients*

| Parameter | Two-drug | Three-drug | Total |
|---|---|---|---|
| Age, years | | | |
| <12 | 2 | 0 | 2 |
| 12–16 | 5 | 5 | 10 |
| >16 | 17 | 8 | 25 |
| Sex | | | |
| Male | 15 | 7 | 22 |
| Female | 9 | 6 | 15 |
| Karnofsky PS | | | |
| 80–100 | 17 | 8 | 25 |
| 60–70 | 6 | 5 | 11 |
| Unknown | 1 | 0 | 1 |
| Alkaline phosphatase | | | |
| Normal | 1 | 7 | 8 |
| Elevated | 23 | 5 | 28 |
| Unknown | 0 | 1 | 1 |
| Primary sites | | | |
| Limbs | 23 | 12 | 35 |
| Axial | 1 | 1 | 2 |
| Metastatic sites | | | |
| Lung | 16 | 11 | 27 |
| Bone | 3 | 0 | 3 |
| Liver | 1 | 0 | 1 |
| Skin | 1 | 0 | 1 |
| Lung and bone | 1 | 0 | 1 |
| Lung, bone and nodes | 1 | 0 | 1 |
| Unknown | 1 | 2 | 3 |
| Total | 24 | 13 | 37 |

clinical or radiological response of the primary, one with no change and five with progressive disease were considered inoperable. In the three-drug arm, 10/11 patients had resection of the primary tumor. In six cases, this was performed after two cycles of chemotherapy, as recommended in the protocol. One patient had surgery 2 weeks after the start of the first cycle because of a pathological fracture, and another had early surgery after the HDMTX component of the second cycle because of progressive disease. Surgery was delayed until after the HDMTX component of cycle 3, for administrative reasons in one case, and in the fourth case surgery was delayed until after four cycles in a patient showing a good clinical and radiological response to chemotherapy.

Table 2 illustrates clinical and radiological responses documented for primary tumors and metastases. For the two-drug arm, by clinical evaluation there were two complete responses (CRs) and eight partial responses (PRs) (overall response rate 48%, exact 95% CI 25–71%) in 21 primary tumors (19 assessable) although only three (14%) of these responses were confirmed by radiology (plain radiographs or CT scan). For the three-drug arm, by clinical evaluation there was one CR and three PRs (overall response rate 40%, exact 95% CI 12–74%) in 10 primary tumors (seven assessable), although only one (10%) of these was confirmed by radiology.

As far as metastases were concerned, in the two-drug arm, there was one CR and six PRs in 21 patients with radiologically evaluable metastases (overall response rate 33%, exact 95% CI 14–57%). In contrast, in the three-drug arm, there were three CRs and three PRs in 11 patients with evaluable metastases (overall response rate 55%, exact 95% CI 23–84%). The estimated difference in overall response rate is 21% (in favor of the three-drug arm) with a 95% exact CI ranging from −16% +61%; the observed difference in overall response rate between therapeutic arms is not significant (Fisher exact two-sided test: $p = 0.28$). When the response distribution was compared by the $\chi^2$ test for trend for ordered categorical variables, no significant difference was found between therapeutic arms ($p = 0.13$)

The median follow-up is 8.6 years and 90% of patients have been followed for more than 6 years. Two-year survival figures are 36% and 69% for two- and three-drug arms, respectively, falling to 9% and 52% at 4 years (unadjusted hazard ratio 0.38, 95% CI 0.18–0.77; unadjusted log-rank test $p = 0.008$). However, when the comparison between the treatment arms was adjusted for the imbalance of AP levels (Fig. 1), excluding one patient for whom AP was unknown, the difference in survival was no longer significant (hazard ratio 0.52, 95% CI 0.22–1.23; $p = 0.14$). An 'intent to treat' analysis, including eligible and the two ineligible patients, provides similar hazard ratios and confidence limits.

Three patients are surviving long-term at 7.5, 10.9 and 11.6 years, all of whom were treated on the three-drug arm. In addition, these three patients presented with normal AP and small metastases in the lung as the only site of distant spread. Two of the three had their lung metastases resected. One patient treated on the three-drug arm, who had progressive lung and bone metastases, was lost to follow-up at 1.5 years. A second patient on the two-drug arm, with progression in lung and brain, was lost to follow-up at 1.3 years. All remaining patients have died.

### Discussion

The most striking initial observation in this study was the significantly better survival for patient, with metastatic disease treated on the three-drug arm, and the fact that all three long-term survivors received this regimen. In addition, a higher proportion of patients in the three-drug arm (10/11 vs 10/21) initially considered to be operable went on to have definitive resection of the primary tumor. This apparent superiority of the three-drug arm is in marked contrast with the results of the adjuvant/neo-adjuvant component of the study[6] in which continuous disease-free survival was significantly better ($p = 0.02$) for patients treated on the two-drug arm, with a similar trend for overall survival ($p = 0.10$).

**Table 2.** *Clinical and radiological responses: primary tumors and metastases*

| Site | Treatment | Response | Clinical | | | | | | |
|---|---|---|---|---|---|---|---|---|---|
| | | Radio* | CR | PR | NC | PD | N/A | A | |
| Primary tumor | Two drug | CR | | | | | | | 0 |
| | | PR | 1 | 2 | 1 | | | | 1 |
| | | NC | 1 | 3 | 1 | | | | 5 |
| | | PD | | 1 | 2 | 4 | | | 7 |
| | | N/A | | 2 | 1 | | 2 | | 5 |
| | | A | | | | | | 3 | 3 |
| | | Total | 2 | 8 | 5 | 4 | 2 | 3 | 24 |
| | Three-drug | CR | | | | | | | 0 |
| | | PR | | 1 | | | 1 | | 2 |
| | | NC | | | 2 | | | | 2 |
| | | PD | | | 2 | | | | 2 |
| | | N/A | 1 | 2 | | | 2 | | 5 |
| | | A | | | | | | 2 | 2 |
| | | Total | 1 | 3 | 4 | 0 | 3 | 2 | 13 |
| Metastases | Two-drug | | 1 | 6 | 5 | 9 | 3 | 0 | 24 |
| | Three-drug | | 3 | 3 | 2 | 3 | 2 | 0 | 13 |

*Plain radiographs or CT scan; CR = complete response; PR = partial response; NC = no change; PD = progressive disease; N/A = not assessable; A = no tumor present.

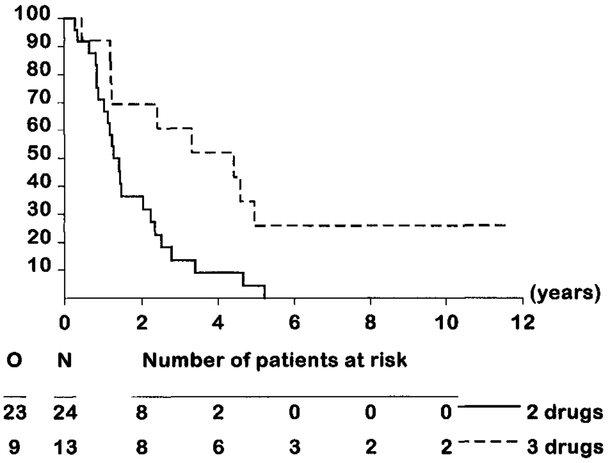

**Fig. 1.** *Overall survival in patients with metastatic osteosarcoma, adjusted according to level of AP at presentation.*

Two important explanations for the discrepancy should be considered:

(1) The difference in effect of the two types of chemotherapy on patients with primary vs metastatic disease has occurred by the play of chance in small populations.

(2) The two types of chemotherapy produced a different biological effect in patients with primary vs metastatic disease.

However, there is a small number of patients, a disparity in the distribution of numbers between the two regimens and an imbalance of known (particularly baseline AP levels) prognostic factors in the metastatic component of the study. It is notable that only one patient in the two-drug arm had a normal AP, whereas seven (58%) had normal levels in the three-drug arm, including all three long-term survivors. Indeed, when adjusted for the imbalance of AP levels between the two therapeutic arms, the difference in survival is no longer apparent.

There is little information in the literature to support the alternative explanation. Many investigators[11–16] believe strongly that HDMTX is an essential component of multi-agent chemotherapy for non-metastatic osteosarcoma and that its efficacy depends on the method of administration. Dose, frequency and concomitant hydration/leucovorin rescue may be crucial, and Delepine *et al.*[17] advocate adaptation of dose based on individual pharmacokinetics. However, patients in both the adjuvant and metastatic components of this study received similar treatment on the three-drug arm.

Different degrees of histological response between 26 primary tumors and concurrent lung metastases resected after a more intensive regimen containing the same three drugs as in the current study have been reported by Bacci *et al.*[18] although discordant responses were observed in only 15% of cases and both primary tumors and metastases showed a similar rate (25%) of good responses. However, there does not seem to be any comparative data describing the effects of different chemotherapy regimens on primary tumors vs metastases.

Our results confirm those of others that the prognosis of patients with metastases at presentation is extremely poor. Only 3/37 (8%) were long-term survivors in this series. Pacquement *et al.*[3] reported long-term outcome for 73 patients receiving a variety of chemotherapy regimens given by members of the French Society of Pediatric Oncology, between 1980 and 1990. At a median 7 years follow-up, 13 patients (18%) were alive and disease free. Similarly, only 7/62 (11%) such patients treated at Memorial Sloan Kettering Cancer Center 1975–1984 with five chemotherapy regimens (T4, T5, T7, T10, T12) were alive at a minimum of 8 years follow-up.[4]

In conclusion, it is likely that random bias in the population (small numbers, imbalance in size of the groups, uneven distribution of AP) accounts for the apparent difference in outcome favouring the three-drug treatment. More reliance can be placed on the finding that disease-free and overall survival in the adjuvant study[6] were better after two-drug treatment.

**Acknowledgements**

Supported by Grants 2U10 CA 11488-13 and 2U10 CA 11488-25 from the National Cancer Institute (Bethesda, Maryland, USA). The UKCCSG acknowledges support from the Cancer Research Campaign, UK. The European Osteosarcoma Intergroup comprises Medical Research Council, UK; Childrens Cancer Study Group. UK; European Organization for Research and Treatment of Cancer.

### References

1 Carter SK. The dilemma of adjuvant chemotherapy for osteogenic sarcoma. *Cancer Clin Trials* 1980; 3:29–36.
2 Bramwell VHC. The role of chemotherapy in osteogenic sarcoma. *Crit Rev Oncol Hematol* 1995; 20:61–85.
3 Pacquement H, Blanchard C, Kalifa C, *et al.* Metastatic osteogenic sarcoma at diagnosis. Study of 73 cases treated by the French Society of Pediatric Oncology (SFOP) between 1980 and 1990. *Proc Am Soc Clin Oncol* 1995; 14:456.
4 Meyers PA, Heller G, Healey JH, *et al.* Osteogenic sarcoma with clinically detectable metastasis at initial presentation. *J Clin Oncol* 1993; 11:449–53.
5 Bramwell VHC. Chemotherapy of operable osteosarcoma. *Bailliere's Clinical Oncology* 1987; 1:175–203.
6 Bramwell VHC, Burgers M, Sneath R, *et al.* A comparison of two short intensive adjuvant chemotherapy regimens in operable osteosarcoma of limbs in children and young adults: the first study of the European Osteosarcoma Intergroup. *J Clin Oncol* 1992; 10:1579–91.
7 Bleyer WA. Methotrexate: clinical pharmacology, current status and therapeutic guidelines. *Cancer Treat Rep* 1977; 4:87–101.
8 Sylvester R. On the analysis of response rates in studies of advanced disease. *Eur J Cancer* 1980; 1:5–7.
9 Kaplan EL, Meier P. Nonparametric estimation from incomplete observations. *J Am Stat Assoc* 1958; 53:457–81.
10 Parmar MKB, Machin D. *Survival analysis: a practical approach.* Chichester: John Wiley & Sons, 1995.
11 Jaffe N, Frei E, Traggis D, *et al.* Weekly high-dose methotrexate citrovorum factor in osteogenic sarcoma. *Cancer* 1977; 39:45–50.
12 Jaffe N, Frei E, Watts H, *et al.* High-dose methotrexate in osteogenic sarcoma: a 5 year experience. *Cancer Treat Rep* 1978; 62:259–64.
13 Rosen G, Nirenberg A. Chemotherapy for osteogenic sarcoma: an investigative method, not a recipe. *Cancer Treat Rep* 1982; 66:1687–97.
14 Winkler K, Beron G, Delling G, *et al.* Neoadjuvant chemotherapy of osteosarcoma: results of a randomized cooperative trial (COSS-82) with salvage chemotherapy based on histological tumor response. *J Clin Oncol* 1988; 6:329–37.
15 Bacci G, Picci P, Ruggieri P, *et al.* Primary chemotherapy and delayed surgery (neoadjuvant chemotherapy) for osteosarcoma of the extremities. *Cancer* 1990; 65:2539–53.
16 Graf N, Winkler K, Betlemovic M, *et al.* Methotrexate pharmacokinetics and prognosis in osteosarcoma. *J Clin Oncol* 1994; 12:1443–51.
17 Delepine N, Delepine G, Cornille H, *et al.* Dose escalation with pharmacokinetics monitoring in methotrexate chemotherapy of osteosarcoma. *Anticancer Res* 1995; 15:489–94.
18 Bacci G, Picci P, Briccoli A, *et al.* Osteosarcoma of the extremity metastatic at presentation: Results achieved in 26 patients treated with combination therapy (primary chemotherapy followed by simultaneous resection of the primary and metastatic lesions). *Tumori* 1992; 78:200–6.

### Appendix

The following physicians contributed patients to this European Osteosarcoma Intergroup study.

*United Kingdom: (data submitted to MRC Cancer Trials Office, Cambridge).* R. Souhami, London Bone Tumour Service, London; R. Sneath, R. Grimer, The Royal Orthopedic Hospital, Birmingham; J. Martin, Alder Hay Children's Hospital, Liverpool; V. Bramwell, D. Crowther, Christie Hospital, Manchester; J. Bullimore, Bristol Radiotherapy Centre, Bristol; J. Green, Clatterbridge Hospital, Merseyside; R. Leonard, Western General Hospital, Edinburgh; O. Eden, Royal Hospital for Sick Children, Edinburgh; G. Rustin, Mt Vernon Hospital, Northwood; R. Evans, Newcastle General Hospital, Newcastle-upon-Tyne; J. Stewart, Northampton General Hospital, Northampton.

*Continental Europe: (data submitted to EORTC Data Centre, Brussels).* A. Tamineau, A. van Oosterom, Akademisch Ziekenhaus, Leiden, The Netherlands; J. Rouesse, Institut Gustav Roussy, Villejuif, France; R. Somers, M. Burgers, Antoni van Leuwenhoek Ziekenhaus, Amsterdam, The Netherlands; P. Voute, Emma Kinderziekenhuis, Amsterdam. The Netherlands; T. Wagener, St Radboud Ziekenhaus, Nijmegen, The Netherlands; P. Lucas, Institut Jean Godinot, Reims, France; C. Mendiola, Hopital 1° de Octobre, Madrid, Spain.
